# Centrosome maturation requires YB-1 to regulate dynamic instability of microtubules for nucleus reassembly

**DOI:** 10.1038/srep08768

**Published:** 2015-03-05

**Authors:** Atsushi Kawaguchi, Masamitsu N. Asaka, Ken Matsumoto, Kyosuke Nagata

**Affiliations:** 1 Department of Infection Biology, Faculty of Medicine, University of Tsukuba, 1-1-1 Tennodai, Tsukuba 305-8575, Japan; 2 Chemical Genetics Laboratory, RIKEN, 2-1 Hirosawa, Wako, Saitama 351-0198, Japan

## Abstract

Microtubule formation from the centrosome increases dramatically at the onset of mitosis. This process is termed centrosome maturation. However, regulatory mechanisms of microtubule assembly from the centrosome in response to the centrosome maturation are largely unknown. Here we found that YB-1, a cellular cancer susceptibility protein, is required for the centrosome maturation. Phosphorylated YB-1 accumulated in the centrosome at mitotic phase. By YB-1 knockdown, microtubules were found detached from the centrosome at telophase and an abnormal nuclear shape called nuclear lobulation was found due to defective reassembly of nuclear envelope by mis-localization of non-centrosomal microtubules. In conclusion, we propose that YB-1 is important for the assembly of centrosomal microtubule array for temporal and spatial regulation of microtubules.

The microtubule cytoskeleton is required for spatially and temporally controlled dynamics of diverse cellular processes, including remodeling of cellular organelle, formation of mitotic spindle, and protein trafficking. The architecture of the microtubule array is formed depending on not only the dynamic instability of microtubules but also microtubule nucleation and anchoring at the centrosome, which is the main microtubule-organizing centre (MTOC) in proliferating animal cells. The centrosome is composed of a pair of centrioles surrounded by more than a hundred different proteins including γ-tubulin ring complex (γ-TuRC), a multi-subunit protein complex containing γ-tubulin required for the microtubule nucleation^1^,^2^. The level of γ-TuRC at pericentriolar region increases dramatically prior to mitosis concomitantly with recruitment of microtubule-associated proteins which are required for mitotic spindle formation under the control of Polo-like and Aurora A kinases^3^. This process is termed centrosome maturation. However, the precise mechanism of microtubule assembly including factors responsible for the centrosome maturation is not fully understood.

Nuclear envelope (NE) is a cellular structure that encloses chromosomes and provides a framework for gene expression and DNA replication. NE consists of inner and outer membranes that are joined by the nuclear pore complexes (NPC). The outer membrane is continuous with ER, and the nuclear lamina, a meshwork composed of nuclear lamin proteins, underlies the inner membrane. The re-assembly of NE at telophase might be crucial to re-establish a functional nucleus for the next interphase. It is proposed that NE re-assembly begins with attachment of precursor membranes to telophase chromosomes, followed by fusion of the membranes and re-assembly of NPC and nuclear lamina into NE^4^. It is thought that targeting of precursor membranes to the chromosomal surface could be mediated by chromatin-binding membrane proteins such as LAP2β and lamin B receptor (LBR). Alternatively, BAF, a chromatin-binding protein, is also required for the re-assembly of NE by sequentially recruiting precursor membranes via its direct interaction with LEM domain-containing nuclear membrane proteins, LAP2α, emerin, and MAN1, respectively. However, the detail mechanism of membrane transport mediated by theses NE proteins at telophase is still unknown.

In the nucleus, Y-box binding protein-1 (YB-1) functions as a transcription factor and splicing regulator^5^. However, YB-1 is mainly localized in the cytoplasm and regulates translation and stability of mRNA as a major component of cellular mRNA ribonucleoprotein^6^. Therefore, it is proposed that YB-1 determines the fate of cellular mRNAs from their synthesis to destruction. YB-1 is overexpressed in over 75% of human breast carcinomas, and its amount is shown to correlate with breast cancer aggressiveness^7^. It is also reported that YB-1 accumulates in the centrosome during G2/M phases in a phosphorylation-dependent manner^8^. Further, ectopically overexpressing YB-1 provokes remarkably diverse breast carcinomas through the induction of genetic instability caused by the mitotic failure and centrosome amplification^9^. Therefore, YB-1 is postulated as a cancer susceptibility gene with the capacity to prime cells for tumorigenesis by regulating the centrosome function, although the detail mechanism is not fully clear^8^,^10^.

Here, we found that YB-1 is required for the centrosome maturation. In YB-1 knockdown (KD) cells, lobulated nuclei were assembled at G1 phase due to a defective reassembly of nuclear envelope (NE) caused by a sporadic non-centrosomal microtubule formation at the end of mitosis. We propose that YB-1 is important for the temporal and spatial regulation of microtubules to establish centrosomal microtubules for the re-assembly of NE.

## Results

### YB-1 is required for the centrosome maturation at mitosis

**Figure 1:**
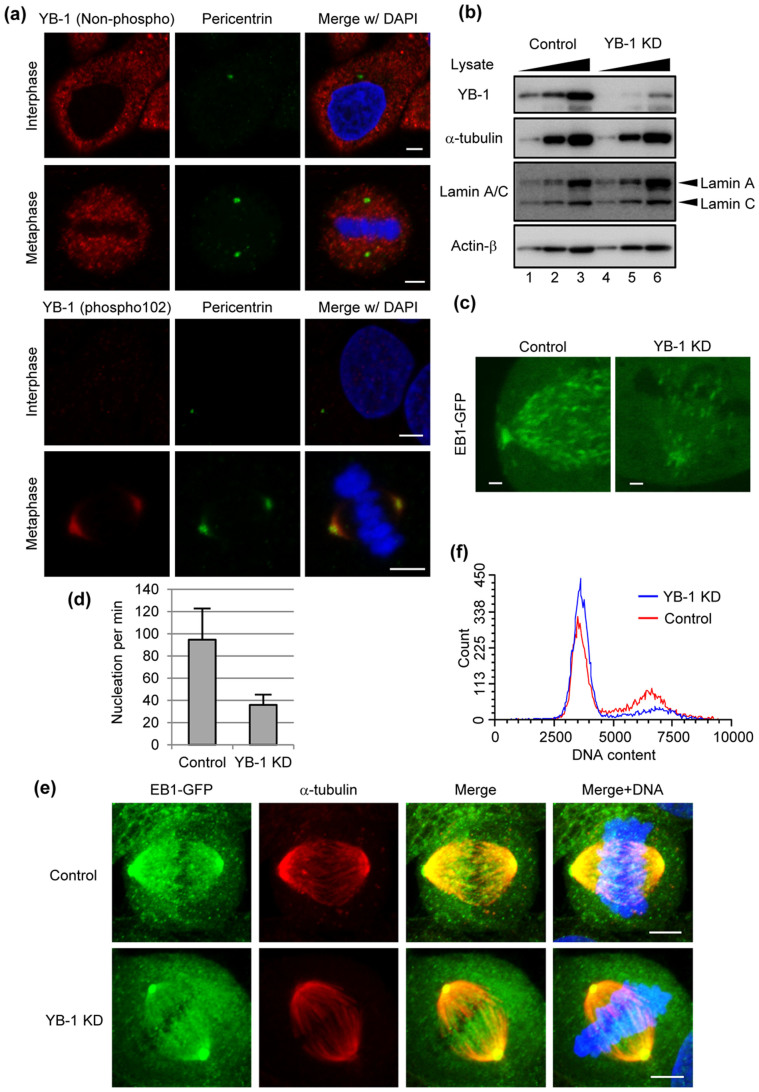
Metaphase progression of YB-1 KD cells. (A) HeLa cells were subjected to indirect immunofluorescence assays with mouse anti-pericentrin (green) and either rabbit anti-non-phosphorylated YB-1 (red; upper panels) or rabbit anti-YB-1 phospho Ser102 (red; lower panels) antibodies, respectively. Scale bar, 5 μm. (B) HeLa cells were transfected with either non-targeting (control; lanes 1–3) or YB-1 siRNA (YB-1 KD; lanes 4–6). After 48 h post transfection, the cells were lysed, and the lysates (5 × 10^3^, 1 × 10^4^, and 2 × 10^4^ cells) were analyzed by SDS-PAGE followed by western blotting assays with anti-YB-1, anti-α-tubulin, anti-lamin A/C, and anti-actin-β antibodies. Full-length blots are presented in Supplementary Fig. 1. (C) At 48 h post transfection with either non-targeting (control) or YB-1 siRNA (YB-1 KD), HeLa cells were subjected to the live cell imaging using confocal microscopy. Images of mitotic centrosome were acquired at 1.57-sec intervals for 1 min (see also Supplementary Movie S1 and S2). The snap shots taken from the time-lapse image are shown. Scale bar, 1 μm. (D) EB1-GFP comets emerged from the centrosome were counted by IMARIS 7.2 software (Carl Zeiss). The results are averages from three independent experiments with standard deviations (*n* = 5). (E) At 48 h post transfection of either non-targeting (control) or YB-1 siRNA (YB-1 KD), HeLa-EB1-GFP cells were fixed and subjected to the indirect immunofluorescence assays with rabbit anti-GFP (green) and mouse anti-α-tubulin (red) antibodies. Images of mitotic cells were acquired. Scale bar, 5 μm. (F) At 48 h post transfection of either non-targeting (control) or YB-1 siRNA (YB-1 KD), the cells were trypsinized and fixed in 70% ethanol. After RNase A treatment, cells were stained with propidium iodide, and subjected to FACS analysis.

**Figure 2:**
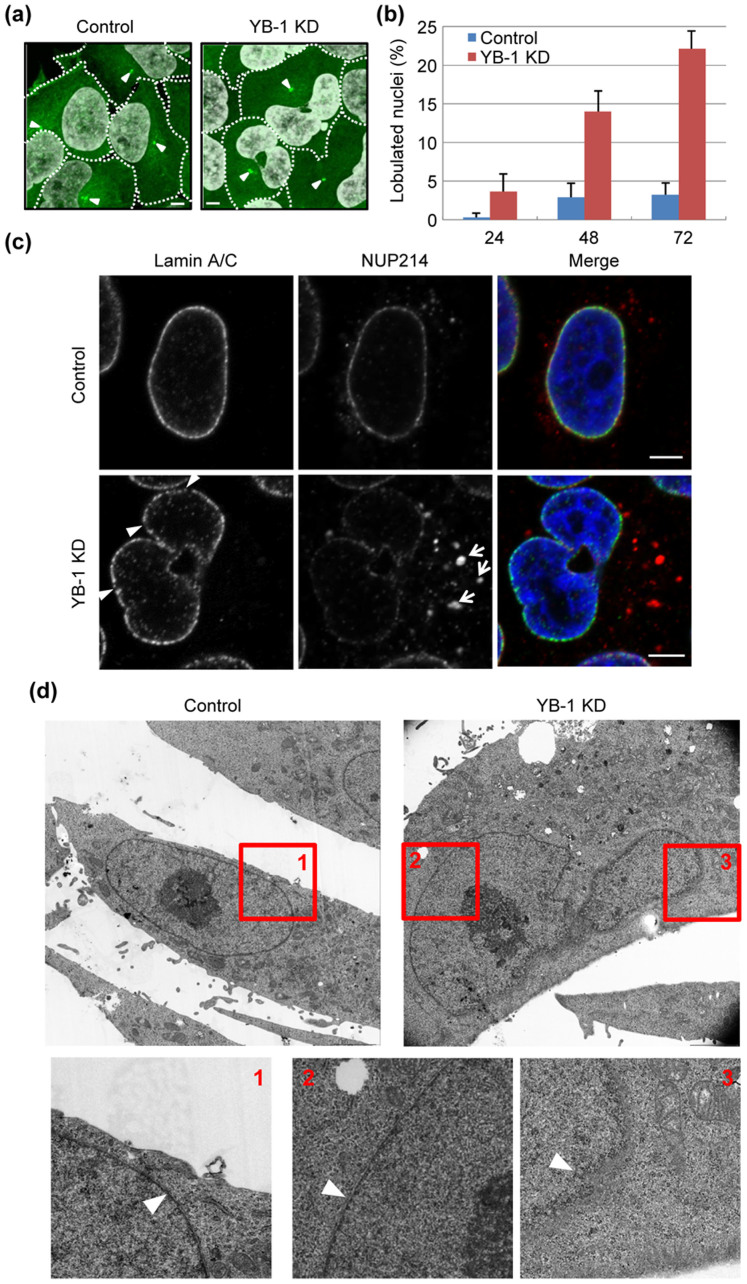
Nuclear lobulation of YB-1 KD cells. (A, B) At 48 h post transfection with either non-targeting (control) or YB-1 siRNA (YB-1 KD), HeLa-EB1-GFP cells were stained with DAPI. The arrowheads indicate EB1-GFP accumulating in the centrosome. Dotted lines indicate the outline of each cell. Scale bar, 5 μm. The ratio of lobulated nuclei to the total cell number is indicated in Fig. 2B. The data represent mean values with standard deviations from three independent experiments (n > 250). (C) At 48 h post transfection with either non-targeting (control) or YB-1 siRNA (YB-1 KD), HeLa cells were subjected to the indirect immunofluorescence assay with anti-lamin A/C (green) and NUP214 (red) antibodies, respectively. The annulate lamellae and the discontinuous regions of nuclear lamina are indicated by arrows and arrowheads, respectively. Scale bar, 5 μm. (D) At 48 h post transfection with either non-targeting (control) or YB-1 siRNA (YB-1 KD), HeLa cells were subjected to transmission electron microscopic analysis. Nuclear envelope is indicated by arrowheads.

It is reported that YB-1 is phosphorylated at G2/M phases and then localized to the centrosome^8^ as shown in Fig. 1A. Since the microtubule nucleating capacity is increased at the onset of mitosis^11^, it is assumed that YB-1 is involved in the microtubule formation during mitosis. To address this, we examined the microtubule nucleation at metaphase using cells constitutively expressing EB1-GFP^11^, which specifically interacts with growing microtubule ends. The expression level of YB-1 in KD cells decreased to approximately 10% of that in control cells (Fig. 1B). The time series of EB1-GFP were acquired at 1.56-sec intervals for 1 min. The amount of EB1-GFP comets nucleated from the centrosome was significantly reduced in YB-1 KD cells at metaphase (Fig. 1C, D, Supplementary Movie 1, 2). Thus, it is likely that the translocation of YB-1 to the centrosome is required for the centrosome maturation. However, the spindle assembly during metaphase was unaffected in the absence of YB-1 (Fig. 1E), and YB-1 KD cells did not show any aneuploidy (Fig. 1F). It has been reported that the spindle assembly and mitosis can occur in cells, in which the functional centrosome is not present, similar to other organisms lacking centrosome such as plant^12^,^13^,^14^,^15^. This is due to the fact that the chromatin-mediated pathway of spindle assembly obscures the defect of microtubule growth from the centrosome^16^. These studies also suggest that the loss of centrosome is a stress signal that leads cells to G1 arrest. Similarly, YB-1 KD cells were arrested in G1 phase but not mitotic phase (Fig. 1F). However, it is reported that a number of genes involved in the cell cycle progression, e.g. *CCNA1*, *CCNB1*, and *CDC6*, might be transcriptionally regulated by YB-1^17^,^18^. Further analyses are required to reveal the role of YB-1 in the cell cycle progression. In addition, we also found that approximately 20% of YB-1 KD cells formed lobulated nuclei at 48 h post transfection of siRNA (Fig. 2A, B).

### Defective nuclear envelope reassembly in YB-1 KD cells at telophase

Most cells have ovoid or spherical shaped nuclei. The lobulated nuclei are hardly observed except for the aged cells or certain highly mobile cell types such as myeloid and cancer cells^19^. As seen in a case of premature aging Hutchison-Gilford progeria syndrome (HGPS), an altered nuclear shape is mainly due to changes in nuclear lamina^20^. To address the integrity of NE in YB-1 KD cells, we observed intracellular localization of lamin A/C and NUP214, which is one of NPC proteins. The expression level of lamin A/C in YB-1 KD cells was similar to that in control cells (Fig. 1B). However, we observed discrete localization of lamin A/C and NUP214 at the nuclear membrane in YB-1 KD cells (Fig. 2C). Further, we carried out transmission electron microscopic analyses (Fig. 2D). Nuclear envelope is indicated by arrowheads. In enlarged panels 1 and 2, nuclear envelopes were found to be intact. In contrast, a part of nuclear envelope was locally thinned and difficult to find in YB-1 KD cells as shown in enlarged panel 3. In addition, a part of NUP214 was found as punctate signals in the cytoplasm (Fig. 2C, arrows). These cytoplasmic signal of NUP214 might be an NPC assembled onto the ER membrane called “annulate lamellae”^21^. The annulate lamellae is induced by prolonged exposure to sub-lethal doses of microtubule inhibitors, colchicine or vinblastine sulfate^22^, possibly due to a defect of NE reassembly at the end of mitosis. Therefore, it is reasonable to postulate that the intracellular trafficking of the nuclear membrane is impaired in YB-1 KD cells at the end of mitosis.

**Figure 3:**
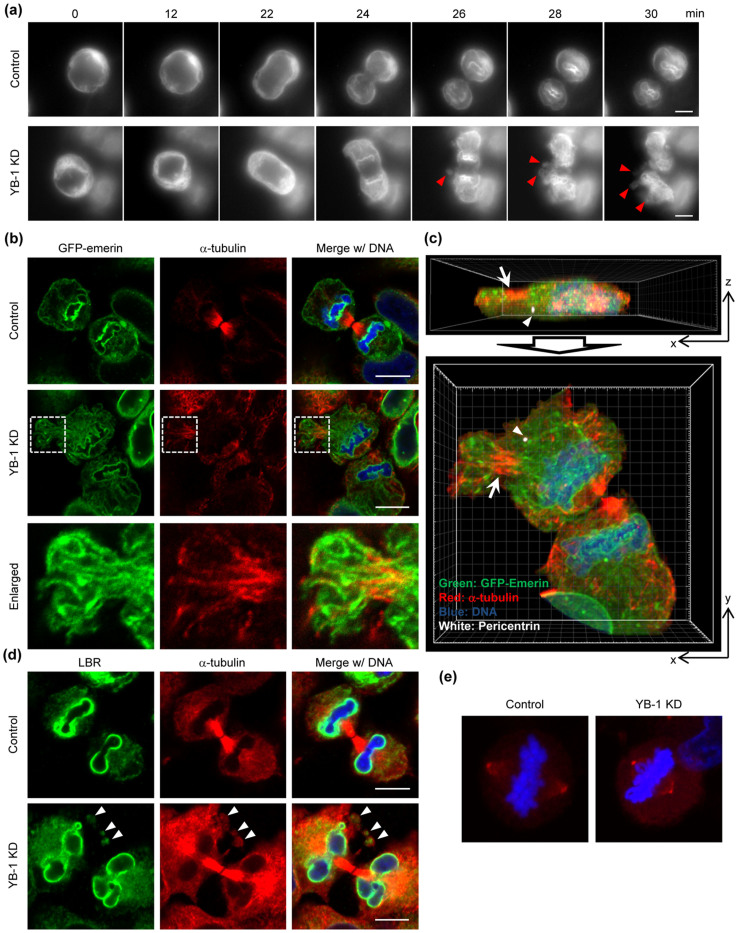
Defect in NE reassembly at telophase in YB-1 KD cells. (A, B, C) At 48 h post transfection with either non-targeting (control) or YB-1 siRNA (YB-1 KD), HeLa-GFP-emerin cells were subjected to the live cell imaging using AxioCam MRm camera (Carl Zeiss; Fig. 3A) and the indirect immunofluorescence assays with anti-α-tubulin (Fig. 3B, C) and anti-pericentrin (Fig. 3C) antibodies. For live-cell imaging, images were acquired at 2-min intervals for 90 min (see also Supplementary Movie S3 and S4), and the sequential images of each movie are shown in Fig. 3A. In Fig. 3B, areas in white boxes are enlarged. Fig. 3C represents the projection images of xz- (upper panel) and xy-sections (lower panel) of YB-1 KD cells shown in Fig. 3B. Image processing was performed using IMARIS 7.2 software (Carl Zeiss). The cells adjacent to the telophase cell were omitted from images. Arrowheads and arrows indicate the centrosome (white) and non-centrosomal microtubules (red), respectively. Nuclei were counter-stained with DAPI (blue). Scale bar, 10 μm. (D) At 48 h post transfection with either non-targeting (control) or YB-1 siRNA (YB-1 KD), HeLa cells were subjected to the indirect immunofluorescence assay with anti-LBR (green) and anti-α-tubulin (red) antibodies. Nuclei were counter-stained with DAPI (blue). The spore-like structures are indicated by arrowheads. Scale bar, 10 μm. (E) At 48 h post transfection with either non-targeting (control) or YB-1 siRNA (YB-1 KD), HeLa cells were subjected to the indirect immunofluorescence assay with anti-γ-tubulin antibody (red). Nuclei were counter-stained with DAPI (blue).

The targeting of precursor membranes to the chromosomal surface is mediated by nuclear membrane proteins such as BAF-emerin complex, LBR, and LAP2β. Based on these, we tried to visualize GFP-emerin to analyze the intracellular trafficking of nuclear membrane at telophase in the presence or absence of YB-1 (Fig. 3A and Supplementary Movie 3, 4). In control cells, a major portion of GFP-emerin was rapidly recruited to the so-called ‘core' regions of segregated chromosomes at telophase (24–28 min) and then evenly distributed around the chromosomes (Supplementary Movie 3, after 30 min) as reported previously^23^. In contrast, a part of GFP-emerin sporadically formed spore-like structures in the peripheral cytoplasm at the end of telophase in YB-1 KD cells (26–30 min, arrowheads), and finally GFP-emerin was integrated to NE in a delayed fashion concomitantly with the chromatid decondensation (Supplementary Movie 4). To further analyze in detail, we examined the intracellular localization of GFP-emerin and microtubules in paraformaldehyde-fixed YB-1 KD cells (Fig. 3B). Similar to the results of Figure 3A, the spore-like structures were found in YB-1 KD cells (Fig. 3B, middle panel), suggesting that the mis-localization of GFP-emerin was not due to cellular damages by light exposure in the live-cell imaging. In addition, GFP-emerin was also found as fibrillar structures along sporadically assembled microtubules (Fig. 3B, enlarged figure; Fig. 3C, arrow) apart from the centrosome (Fig. 3C, arrowhead). Further, we examined intracellular localization of LBR, which is an inner nuclear membrane protein possessing direct chromatin-binding activity, as another marker protein of NE (Fig. 3D). Not only emerin but also LBR was retained in the spore-like structures in YB-1 KD cells. Note that we did not find the chromosomes in the spore-like structures and any multipolar spindles in YB-1 KD cells (Fig. 3E), thus the chromosome partitioning might occur correctly.

### Microtubule destabilizing reagent improves the nuclear lobulation caused by YB-1 KD

**Figure 4:**
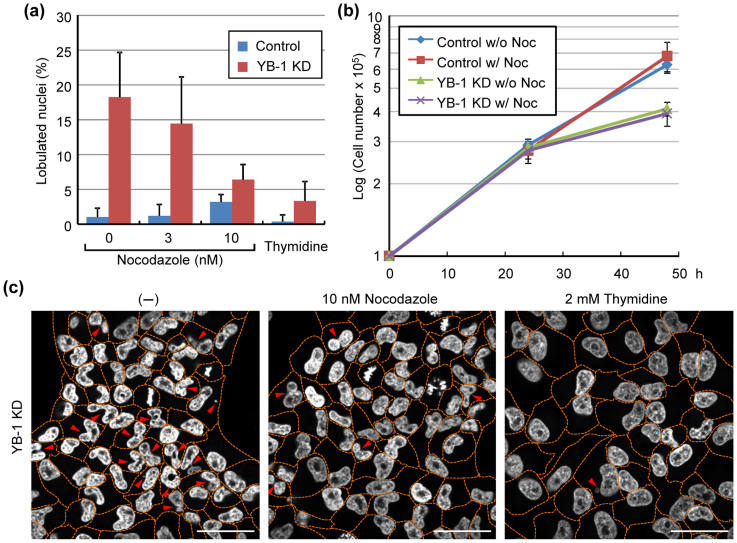
Low Concentration of Nocodazole Attenuates the Formation of Lobulated Nuclei in YB-1 KD Cells. At 12 h post transfection with either non-targeting (control) or YB-1 siRNA (YB-1 KD), the cells were treated with either 3 nM, 10 nM nocodazole, or 2 mM thymidine for 36 h. (A) At 48 h post transfection, the number of lobulated nuclei was counted. The data represent mean values with standard deviations from three independent experiments (n > 250). (B) The number of cells was determined in the absence or presence of 10 nM nocodazole at 24 and 48 h post transfection, respectively. (C) Representative results summarized in Fig. 4A are shown. Nuclei were stained with DAPI. Dotted lines indicate the outline of each cell. The lobulated nuclei or micro nuclei are indicated by arrowheads. Scale bar, 50 μm.

It is assumed that mis-localization of non-centrosomal microtubules impairs the reassembly of NE at the onset of G1 phase. To prove this, we examined the nuclear lobulation in cells treated with a low concentration of nocodazole (Fig. 4). At 12 h post transfection of siRNA, HeLa cells were treated with 10 nM nocodazole and then further incubated for 36 h. The effect of the low concentration of nocodazole used here on the cell growth was not found in HeLa cells as shown in Fig. 4B. The lobulated nuclei in YB-1 KD cells were significantly reduced by the addition of nocodazole (Fig. 4A, C). Further, when we treated YB-1 KD cells with 2 mM of thymidine to arrest the cells in G1 phase, the lobulated nuclei were hardly appeared (Fig. 4A, C). Therefore, it is quite likely that the anchoring of microtubules to the centrosome mediated by YB-1 is important for the temporal regulation of microtubule dynamics during mitosis for the proper reassembly of NE in the next G1 phase.

## Discussion

It has been reported that YB-1 is localized in the centrosome during mitosis^8^, but its functional details in the centrosome are not known. It is shown that several proteins including pericentrin, ninein, Nlp, CDK5RAP2, and GCP-WD/NEDD1 are implicated in the assembly of microtubules from the centrosome^24^,^25^,^26^,^27^,^28^. However, little is known about the microtubule assembly in response to the centrosome maturation. Our results strongly support the notion that YB-1 is required for the formation of microtubule from the centrosome in metaphase (Fig. 1C, D).

In most proliferating animal cells, the centrosome is the main organelle for microtubule nucleation and anchoring, leading to the formation of radial microtubule arrays. The minus ends of microtubules are anchored at the centrosome, while the growing plus ends are stabilized by the plus-end tracking proteins (+TIPs) such as EB1 and APC^29^. However, in differentiated cells such as muscle, epithelial, and neuronal cells, a major portion of microtubules are non-centrosomal and usually linear^30^. In this case, non-centrosomal microtubules are translocated to the peripheral regions possibly using microtubule-dependent motor proteins or treadmilling^31^,^32^. After the translocation, non-centrosomal microtubules are captured to the cell cortex mediated by interactions between +TIPs and actin filaments^33^, and are stabilized by bundling into linear microtubule arrays^30^. In mitosis, the microtubule dynamics are tightly controlled by both stabilizers and destabilizers of the microtubule plus ends such as MCAK mitotic kinesin to achieve accurate chromosome segregation and spindle positioning^34^,^35^. Since YB-1 is required for the assembly of centrosomal microtubules (Fig. 1C, D), it is possible that the sporadically accumulated non-centrosomal microtubules are hardly disassembled at an appropriate time during telophase in YB-1 KD cells as shown in Fig. 3.

At the onset of mitosis, microtubules interact with NE mediated by a dynein motor protein for the nuclear envelope break down (NEBD) by rupturing the nuclear membrane^36^,^37^. After NEBD, nuclear membrane and its associated proteins are re-distributed into mitotic ER, which is a highly inter-connected network of tubular structures^38^. We found that the non-centrosomal microtubules were sporadically accumulated in the peripheral regions of YB-1 KD cells at telophase together with fibrillar structures of GFP-emerin (Fig. 3B, C). Therefore, in YB-1 KD cells, it is possible that the reassembly of NE on the chromosomes is inhibited through capturing of the mitotic ER to non-centrosomal microtubules, which are aberrantly stabilized at the cell cortex. It has also been reported that low concentrations of paclitaxel, which is a potent stabilizer of microtubules, induces the lobulated nuclei at next G1 phase after mitosis^39^. This is in good agreement with the results of Figure 4. Thus, we propose that the temporal control of microtubule disassembly at telophase is achieved by assembling centrosomal microtubules in an YB-1-dependent manner to release nuclear membrane from microtubules.

The recruitment of nuclear membrane to DNA depends on the high DNA affinity of the multiple inner nuclear membrane proteins, such as LAP2β, LBR, and BAF-emerin complex^40^. Following the initial binding process, the tubular precursor membrane is flatten into sheets and then spread across the chromatin and re-organized into a sealed NE^41^. Once the sealed NE with functional NPCs is formed, NE expands to its final size and shape concomitantly with the de-condensation of chromatids. As shown in Fig. 3 and Supplementary Movie 4, it is suggested that, in YB-1 KD cells, the accumulation of precursor membranes on the chromosomes tends to be delayed and NE cannot be reassembled completely before starting the chromatid de-condensation. It is proposed that when the nuclear membrane is assembled around a subset of chromosomes by KD of lamin proteins, the multiple and/or lobulated nuclei are generated in G1 phase^42^. Therefore, it is likely that the proper nuclear formation requires the temporal and spatial regulation of intracellular trafficking of nuclear membrane onto the chromosome in telophase. The linker of nucleoskeleton and cytoskeleton (LINC) complex proteins are believed to tether the interphase NE to various cytoskeletal elements^43^. YB-1 did not accumulate in the centrosome during interphase (Fig. 1), and the lobulated nuclei were not found in YB-1 KD cells arrested in G1 phase by the addition of thymidine (Fig. 4). These suggest that YB-1 regulates the nuclear formation in a LINC complex-independent manner.

YB-1 is overexpressed in over 75% of human breast carcinomas, and its amount is shown to correlate with breast cancer aggressiveness^7^. Recently it is reported that YB-1 determines the chemosensitivity of tumor cells to paclitaxel in a focal adhesion kinase-dependent manner^44^. However, the detailed mechanism is not fully understood. Our findings contribute to understanding the functional regulation of centrosome in response to several stimuli as well as providing with novel aspects to studies on physiological mitotic microtubule dynamics possibly related to cancer chemotherapy.

## Methods

### Biological Materials

A rabbit polyclonal antibody against phosphorylated YB-1 was purchased (Cell Signaling Technology). Mouse antibodies against pericentrin (Abcam), lamin A/C (Cell Signaling Technology), α-tubulin (Sigma), and γ-tubulin (Sigma) and a rabbit polyclonal antibody against NUP214 (Abcam) and lamin B receptor (Abcam) were purchased. HeLa cells were grown in minimal essential medium (MEM) containing 10% fetal bovine serum. For the construction of plasmids expressing GFP-centrin-2, EB1-GFP, and GFP-emerin, HeLa total RNA was reverse-transcribed as a template using oligo(dT)_20_ primer, and the cDNAs were amplified with primers 5′-ATCGTCGACATGGCCTCCAACTTTAAGAAGG-3′ and 5′-GGGAGATCTTTAATAGAGGCTGGTCTTTTTCA-3′ for centrin-2, and 5′-CCCGCTAGCCGCCACCATGGCAGTGAACGTATACTC-3′ and 5′-GGCGCTAGCGGATACTCTTCTTGCTCCTCC-3′ for EB1, and 5′-TTTAAGCTTCCACCATGGACAACTACGCAGATCT-3′ and 5′-AAAGGATCCGCGAAGGGGTTGCCTTCTTCA-3′ for emerin using KOD plus polymerase (TOYOBO). Centrin-2 and EB1 cDNA were cloned into plasmid pCAGGS-GFP, respectively. Emerin was cloned into plasmid pEGFP-C1. To establish HeLa cell lines constitutively expressing either GFP-centrin-2, EB1-GFP, or GFP-emerin, cells were transfected with pSV2-Neo and either pCAGGS-GFP-centrin-2, pCAGGS-EB1-GFP, or pEGFP-emerin. The transfected HeLa cells were selected in the presence of 1 mg/ml of G418 for 2 weeks, and then the G418-resistant colonies were isolated.

### Indirect immunofluorescence assays

Indirect immunofluorescence assays were carried out as previously described^45^. Briefly, cells were fixed with 1% paraformaldehyde (PFA) for 10 min and then pre-permeabilized on ice with 0.01% digitonin in PBS for 5 min. After being washed with PBS, cells were fixed in 4% PFA for 10 min and permeabilized on ice with 0.5% Triton X-100 in PBS for 5 min. After incubation in PBS containing 1% bovine serum albumin for 1 h, coverslips were incubated with each antibody for 1 h and then with Alexa Fluor 488-, 568-, and 633-conjugated secondary antibodies, respectively (Invitrogen). Images were acquired by confocal laser scanning microscopy (LSM700; Carl Zeiss) using ×63 Apochromat objective (NA = 1.4).

### Live-cell imaging

Observations were made with Axio Observer Z1 microscope (Carl Zeiss) using 63× Apochromat objective (NA = 1.4). For examining the intracellular trafficking of NE, images were captured every 2 min for 90 min with AxioCam MRm camera (Carl Zeiss) using a 100 W halogen light source. For tracking of microtubule nucleation from the centrosome, images were acquired at 1.57-sec intervals for 1 min with confocal laser scanning microscopy (LSM700; Carl Zeiss). All experiments were examined at 37°C and 5% CO_2_ in a temperature-controlled stage (Carl Zeiss). Sequential images were processed by using Image J digital image processing software (National Institutes of Bethesda).

### Gene silencing mediated by siRNA

Short interfering RNA against the *YB-1* gene was purchased from Invitrogen. Cells (5 × 10^5^) were transfected with 30 pmol of siRNA using Lipofectamine RNAi Max (Invitrogen) according to the manufacturer's protocol.

### Transmission electron microscope

Cells were fixed with 2.5% glutaralaldehyde. After further fixation with 1% OsO4 for 1 h, sequential dehydrations with ethanol in a step-wise manner were carried out followed by propylene oxide treatment, and embedded in Epon. Ultrathin sections were examined with a JEM-1300 (JEOL) operated at 80 kV.

## Author Contributions

A.K. conceived the research strategies, performed experiments, and wrote the paper. K.N. supervised the research and wrote the paper. A.M. and K.M. prepared plasmids and antibodies.

## Supplementary Material

Supplementary Information Supplementary Movie 1

Supplementary Information Supplementary Movie 2

Supplementary Information Supplementary Movie 3

Supplementary Information Supplementary Movie 4

Supplementary Information Supplementary Information
