# Supplementary material for: Centrosome maturation requires YB-1 to regulate dynamic instability of microtubules for nucleus reassembly
Source: Sci Rep. 2015 Mar 5;5:8768. doi: 10.1038/srep08768 (PMC4350100; doi:10.1038/srep08768)
Supplement: Supplementary Information [file srep08768-s5.pdf]

Title: Centrosome maturation requires YB-1 to regulate dynamic instability of microtubules for nucleus reassembly

Authors: Atsushi Kawaguchi[1*], Masamitsu N. Asaka[1], Ken Matsumoto[2], and Kyosuke Nagata[1]

Addresses:

[1]Department of Infection Biology, Faculty of Medicine, University of Tsukuba, 1-1-1 Tennodai, Tsukuba 305-8575, Japan

[2]Chemical Genetics Laboratory, RIKEN, 2-1 Hirosawa, Wako, Saitama 351-0198, Japan

[*]Correspondence should be addressed to A. K. (e-mail: ats-kawaguchi@md.tsukuba.ac.jp)

Supplemental Legends

Supplementary Movie 1 Live-cell imaging of EB1-GFP in control cells, related to Figure 1C.

At 48 h post transfection of non-targeting siRNA, HeLa-EB1-GFP cells were subjected to live-cell imaging of EB1-GFP nucleated from the centrosome at metaphase. The images were acquired at 1.56-sec intervals for 1 min.

Supplementary Movie 2 Live-cell imaging of EB1-GFP in YB-1 KD cells, related to Figure 1C.

At 48 h post transfection of YB-1 siRNA, HeLa-EB1-GFP cells were subjected to live-cell imaging of EB1-GFP nucleated from the centrosome at metaphase. The images were acquired at 1.56-sec intervals for 1 min.

Supplementary Movie 3 Live-cell imaging of GFP-emerin in control cells, related to Figure 3A.

At 48 h post transfection of non-targeting siRNA, HeLa-GFP-emerin cells were subjected to live-cell imaging at telophase. A major portion of GFP-emerin was rapidly recruited to the segregated chromosomes and then evenly distributed around the chromosomes. Images were acquired at 2-min intervals for 90 min.

Supplementary Movie 4 Live-cell imaging of GFP-emerin in YB-1 KD cells, related to Figure 3A.

At 48 h post transfection of YB-1 siRNA, HeLa-GFP-emerin cells were subjected to live-cell imaging at telophase. A part of GFP-emerin sporadically formed spore-like structures in the peripheral cytoplasm. Images were acquired at 2-min intervals for 90 min.

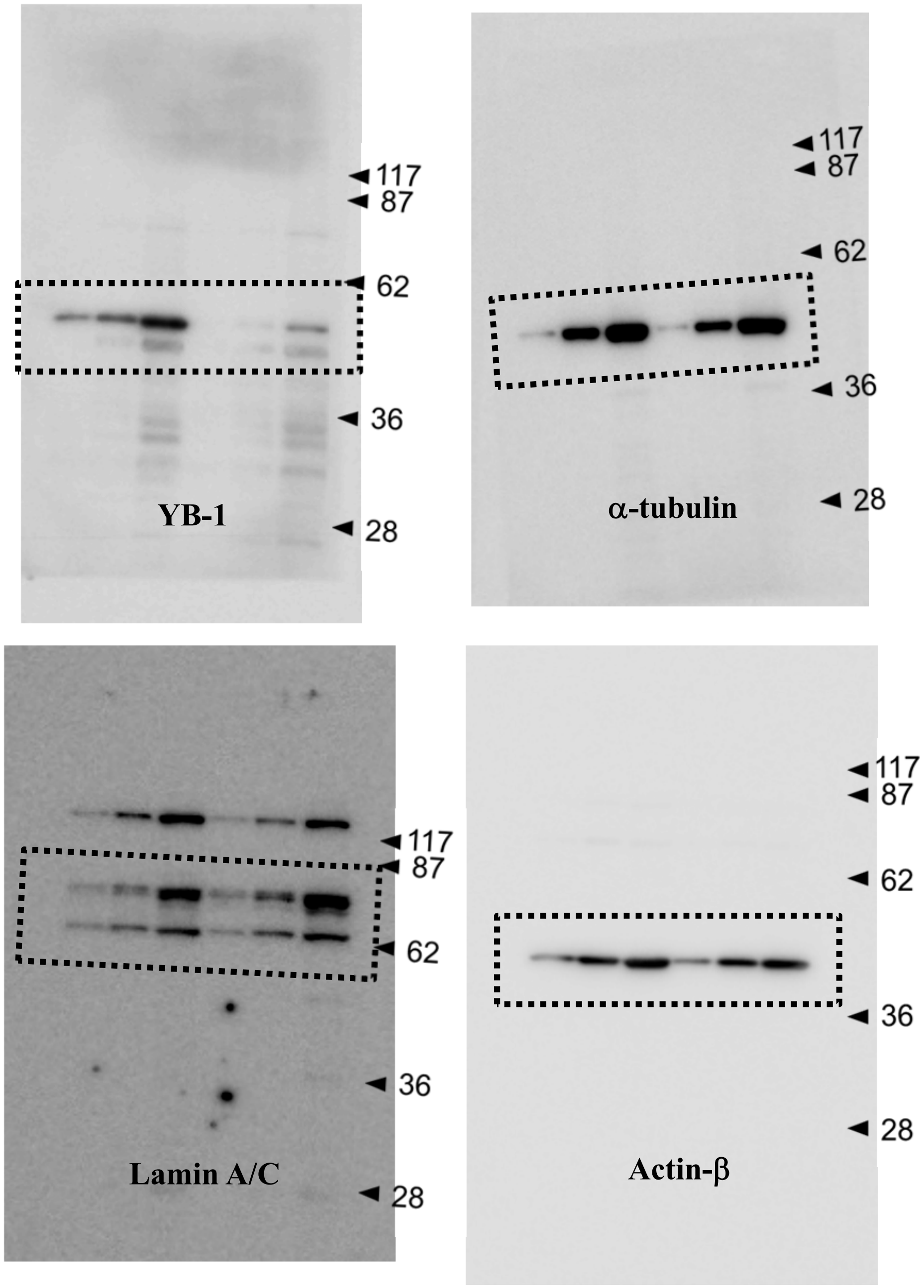

Supplementary Figure 1   Original full-length blots before cropping.

The original blots for Figure 1B.   HeLa cells were transfected with either non-targeting (control; lanes 1-3) or YB-1 siRNA (siYB-1; lanes 4-6).   After 48 h post transfection, the cells were lysed, and the lysates (5×10^3, 1×10^4, and 2×10^4 cells) were analyzed by SDS-PAGE followed by western blotting assays with anti-YB-1, anti-α-tubulin, anti-lamin A/C, and anti-actin-β antibodies.
